# Prevalence and characteristics of e-cigarette users in Great Britain: Findings from a general population survey of smokers

**DOI:** 10.1016/j.addbeh.2014.03.009

**Published:** 2014-06

**Authors:** Jamie Brown, Robert West, Emma Beard, Susan Michie, Lion Shahab, Ann McNeill

**Affiliations:** a Cancer Research UK Health Behaviour Research Centre, University College London, UK; b UK Centre for Tobacco and Alcohol Studies; c National Centre for Smoking Cessation and Training, London, UK; d Department of Clinical, Educational and Health Psychology, University College London, London, UK; e Addictions Department, Institute of Psychiatry, King's College London, UK

**Keywords:** Smoking, Cessation, Quitting, E-cigarettes, Electronic cigarettes

## Abstract

**Background:**

E-cigarettes may be effective smoking cessation aids and their use by smokers has been growing rapidly. It is important to observe and assess natural patterns in the use of e-cigarettes whilst experimental data accumulates. This paper reports the prevalence of e-cigarette awareness, beliefs and usage, including brand choice, and characterises the socio-demographic and smoking profile associated with current use, among the general population of smokers and recent ex-smokers.

**Methods:**

Data were obtained from 3538 current and 579 recent ex-smokers in a cross-sectional online survey of a national sample of smokers in Great Britain in November and December 2012. Differences between current and recent ex-smokers in the prevalence of e-cigarette awareness, beliefs and usage were examined and the socio-demographic and smoking profile associated with current use of e-cigarettes was assessed in a series of simple and multiple logistic regressions.

**Results:**

Ninety-three percent of current and recent ex-smokers (n = 3841) were aware of e-cigarettes. Approximately a fifth (n = 884) were currently using e-cigarettes, whilst just over a third (n = 1507) had ever used them. Sixty-seven percent of the sample (n = 2758) believed e-cigarettes to be less harmful than cigarettes; however, almost a quarter (n = 994) remained unsure. Among both current and recent ex-smokers, the most popular reasons for using were health, cutting down and quitting (each > 80%) and 38% used the brand ‘E-lites’. Among current smokers who were aware of but had never used e-cigarettes, approximately half (n = 1040) were interested in using them in the future. Among current smokers, their use was associated with higher socio-economic status (OR = 1.48, 95%CI = 1.25–1.75), smoking more cigarettes (OR = 1.02, 95%CI = 1.01–1.03) and having a past-year quit attempt (OR = 2.82, 95%CI = 2.38–3.34).

**Conclusions:**

There is a near universal awareness of e-cigarettes and their use appears to be common among smokers in Great Britain although a quarter of all smokers are unsure as to whether e-cigarettes are less harmful than cigarettes. E-lites – a brand that delivers a low dose of nicotine – is the most popular. E-cigarette users appear to have higher socio-economic status, to smoke more cigarettes per day and to have attempted to quit in the past year.

## Highlights

• There is now near universal awareness of e-cigarettes.
• Use is common among smokers.
• Quarter of all smokers unsure as to whether they are less harmful than cigarettes.
• E-lites – a brand that delivers a low dose of nicotine – is the most popular.
• Users have higher SES, smoke more heavily and have attempted to quit recently.

## 1 Introduction

Smoking is one of the leading risk factors for premature death and disability (Lim et al., 2012). The mortality and morbidity associated with cigarette smoking arises primarily from the inhalation of toxins other than nicotine contained within the smoke. By providing a heated vapour containing nicotine without tobacco combustion, electronic cigarettes (e-cigarettes) appear to reduce the cravings and withdrawal symptoms associated with abstinence in smokers (Bullen et al., 2010; Dawkins, Turner, Hasna, & Soar, 2012; Vansickel, Cobb, Weaver, & Eissenberg, 2010) whilst being much safer than ordinary cigarettes (Goniewicz, Knysak, et al., 2013). Moreover, e-cigarettes may be more effective in helping with smoking reduction or cessation than traditional forms of nicotine replacement therapy (NRT) by more closely mimicking the sensory experience and/or nicotine delivery of cigarettes. Two recent randomised controlled trials have suggested that e-cigarettes may aid smoking cessation (Bullen et al., 2013; Caponnetto et al., 2013).

E-cigarettes provide nicotine via a vapour that is drawn into the mouth and upper airways as with cigarettes (Bullen et al., 2010). These devices use a battery-powered heating element to heat a nicotine solution and transform it into vapour. The nicotine is suspended in a mixture of glycerin, propylene glycol or other humectant with water and provided in a cartridge or tank that in some cases are replaceable or refillable (Goniewicz, Knysak, et al., 2013). The process of transforming the solution to vapour is usually activated by the act of inhaling through, or ‘vaping’, the device (Dawkins, Turner, Roberts, & Soar, 2013). The concentration of nicotine delivered to the bloodstream appears to depend upon the experience of users and the brand of e-cigarette (Etter & Bullen, 2011; Vansickel & Eissenberg, 2013). The reason for the latter is likely that different e-cigarette brands and models vary considerably in the efficacy and consistency with which they vaporise nicotine (Goniewicz, Hajek, & McRobbie, 2014; Goniewicz, Kuma, Gawron, Knysak, & Kosmider, 2013).

Evidence to date suggests that e-cigarettes are increasing rapidly in popularity (Dawkins et al., 2012; Dockrell, Morison, Bauld, & McNeill, 2013; Pearson, Richardson, Niaura, Vallone, & Abrams, 2012). An international study (Adkison et al., 2013), which included the United Kingdom (UK), carried out in 2010/11, found high awareness of e-cigarettes, but low levels overall of trial and usage (3% overall, 4% in the UK). However, a population survey of a British sample found that current use more than doubled between the beginning of 2010 and the beginning of 2012 from approximately 3% to 7% (Dockrell et al., 2013). As a result of the speed with which e-cigarette prevalence is evidently increasing, it is important to continue monitoring the situation; the current study provides up-to-date figures on the latest prevalence.

Whilst assessments of the overall popularity of e-cigarettes are valuable, the variability in nicotine vaporisation between e-cigarettes (Goniewicz, Kuma, Gawron, Knysak, & Kosmider, 2013; Goniewicz et al., 2014) means that it is also useful to assess which e-cigarette brands are most prevalent. Such data could inform future real-world assessments of the public health impact of e-cigarettes. Inferences can be drawn on the leading e-cigarette brands by using sales figures from convenience stores, which appear to suggest that in the United States (US) the leading brands are ‘NJOY’, ‘bluCigs’and ‘Logic’ (Esterl, 2013) and in the UK it is E-Lites and Nicolites (Eastwood, 2014). However, industry wide sales numbers are scarce (Esterl, 2013), and with e-cigarettes often purchased online, it is important to assess the popularity of different brands by asking users directly.

Data are limited on usage patterns and characteristics among representative samples of smokers. One population survey of a US sample in 2010 indicated that approximately 11% of current smokers and 2% of former smokers had ever used e-cigarettes and were more likely to be from higher socio-economic groups (Pearson et al., 2012). The international study, referred to above, found that well educated smokers were more likely to be e-cigarette users and their use was also associated with two extremes of smoking frequency — nondaily smokers and heavier smokers (more than 20 cigarettes a day; Adkison et al., 2013).

In view of the growing popularity of e-cigarettes, there is a need for an up-to-date assessment in Great Britain among current and recent ex-smokers of i) beliefs about, awareness and prevalence of e-cigarette use, including different brands, ii) usage patterns of e-cigarettes, and iii) the socio-demographic and smoking characteristics associated with their use.

## 2 Methods

### 2.1 Study design

This was a cross-sectional online survey of a sample of the general population of smokers in Great Britain. The study sample was recruited from an online panel managed by Ipsos MORI. The panel consists of contact details for members of the public who have expressed an interest in taking part in research surveys in exchange for vouchers or entering prize draws. Members of the panel who had smoked in the past-year were invited to complete the full online survey. Approval was granted by the University College London ethics committee.

### 2.2 Participants

During November and December 2012, a total of 23,785 respondents were asked a screening question about their current smoking status of whom 25.9% (n = 6165) had smoked in the past year. This prevalence of past-year smoking was similar to that identified by a face-to-face survey of representative samples of the population in England during 2012 (West & Brown, 2013). Of these 6165 smokers, 4117 provided complete data on all survey items and were included in the current study.

### 2.3 Measures

Smoking status was assessed by asking: ‘Which of the following best applies to you?’: (a) ‘I smoke cigarettes (including hand-rolled) every day’; (b) ‘I smoke cigarettes (including hand-rolled) but not every day’; (c) ‘I do not smoke cigarettes at all but I do smoke tobacco of some kind (e.g. pipe or cigar)’; and (d) I have stopped smoking completely in the last year’. Those responding ‘Yes’ to either (a), (b), or (c) were classified as current smokers and those responding ‘Yes’ to (d) were classified as recent ex-smokers.

Current and recent ex-smokers were subsequently asked questions that assessed gender, age and socio-economic status by the occupationally-based National Statistics Socio-Economic Classification (NS-SEC) self-coding method (Office for National Statistics, 2005). The 8-point NS-SEC classification was dichotomised into ‘high’ (managerial, professional & intermediate occupations) and ‘low’ (routine & manual occupations) socio-economic status. Cigarettes per day, previous attempts to quit smoking, and e-cigarette awareness, beliefs and usage were also assessed (see Appendix A for the questions).

### 2.4 Procedure

As members of an online panel maintained by Ipsos MORI, respondents were invited by email to participate in an online survey about smoking. Respondents were told that by completing the survey they would earn points which could be redeemed against high street vouchers or used to enter a prize draw.

### 2.5 Analysis

The analysis plan was agreed a priori and data were analysed using SPSS 21.0.0.0. Differences between current and recent ex-smokers in socio-demographic and smoking characteristics and in awareness, beliefs and usage relating to e-cigarettes were examined with χ^2^ tests and one-way ANOVAs for categorical and continuous variables respectively. Significant omnibus results were investigated further by post-hoc Sidak-adjusted chi-squared tests and t-tests. To assess smoking and socio-demographic characteristics associated with current use of e-cigarettes, we conducted a series of simple and multiple logistic regressions.

## 3 Results

Of the 4117 past-year smokers included in the current study, 3538 were current smokers and 579 were ex-smokers who had stopped in the past year. Table 1 presents the socio-demographic and smoking characteristics of the sample by smoking status.

Table 2 presents e-cigarette awareness, usage and beliefs by smoking status. In both current and recent ex-smokers, there was an almost universal awareness of e-cigarettes. Approximately a third had ever used e-cigarettes and a fifth currently used them regardless of smoking status. There were no differences between current and recent ex-smokers in their beliefs about the harm of e-cigarettes: in both groups, approximately two thirds believed them to be less harmful than cigarettes whilst almost a quarter were unsure.

Among those who were aware of e-cigarettes but had never used them, approximately half of current smokers were interested in future use, which was significantly more than recent ex-smokers. A substantial minority – just under a fifth – of recent ex-smokers were interested in using them in the future. Among current users of e-cigarettes, there was a difference in the frequency of use by smoking status; recent ex-smokers were more likely to use e-cigarettes daily than current smokers with almost half of recent ex-smokers reporting daily use. The most frequent reasons provided for using e-cigarettes were to improve health, to cut down and to quit smoking; all were endorsed by approximately 80% of current users regardless of smoking status. Recent ex-smokers as compared with current smokers were more likely to report using e-cigarettes for taste but were less likely to use them in order to help with temporary abstinence from smoking conventional cigarettes. For both current and recent ex-smokers, the most popular brand was ‘E-lites’ and almost a fifth ‘did not know’ their current brand.

Table 3 presents the characteristics associated with current use of e-cigarettes in both current and recent ex-smokers. Among current smokers, e-cigarette users were more likely to have higher socio-economic status than non-users. They were also more likely to have made a quit attempt in the past year and to smoke more cigarettes than non-users. Among recent ex-smokers, e-cigarette users reported having previously smoked more cigarettes per day than non-users.

## 4 Discussion

There was a near universal awareness of e-cigarettes among current and recent ex-smokers. Moreover, approximately a fifth of both current and recent ex-smokers were currently using e-cigarettes, whilst just over a third had ever used them. The majority of smokers believed e-cigarettes to be less harmful than cigarettes; however, almost a quarter remained unsure. Among current smokers who were aware of but had never used e-cigarettes, approximately half were interested in using it in the future. Among both current and recent ex-smokers, the most popular reasons for using an e-cigarette were health, cutting down and quitting and the most popular brand was ‘E-lites’. Current e-cigarette users were more likely than non-users to have higher socio-economic status, smoked more cigarettes and attempted to quit in the past year.

The 21% prevalence of current and recent ex-smokers who were using an e-cigarette is considerably higher than the estimates of 3–4% for British and UK samples from 2010 to 2011 (Adkison et al., 2013; Dockrell et al., 2013), and the 7% estimate obtained in a British sample approximately 10 months earlier than the current study (Dockrell et al., 2013). In line with this apparent increase in current use, the prevalence of ever use appears to have increased over those 10 months from 15% to 37%, and awareness from approximately 79% to 93% (Dockrell et al., 2013). The ratio of current to ever use also appeared to have increased across this period, which may suggest that an increasing proportion of smokers who use e-cigarettes are finding that they are sufficiently satisfying or helpful to warrant further use. E-cigarette use is now commonplace in Great Britain and similar to that of licenced NRT (Beard & West, 2012; Kotz, Brown, & West, 2014). This popularity of e-cigarettes combined with their *potential* to be more effective than traditional forms of NRT – as a result of more closely mimicking the sensory experience and/or nicotine delivery of cigarettes – leads to the conclusion that the e-cigarette may have a major public health impact. There is a need for more high quality efficacy studies (Bullen et al., 2013), as well as research to examine the longer-term health impact of use.

There also seems to be scope for the number of people using e-cigarettes to increase: a quarter of all smokers were unsure as to whether e-cigarettes are less harmful than cigarettes and almost half of those who have never used an e-cigarette but were aware of them were interested in using one in the future. However, in the former case, this proportion of people not believing that e-cigarettes are harmful was similar to the estimate obtained at the beginning of 2012 when their use appeared to be considerably lower (Dockrell et al., 2013). The implication is that there may not necessarily be a straightforward decline in the proportion of those believing that e-cigarettes are less harmful as more people begin using them and relative risks of these products probably need to be communicated more widely to smokers.

The relative popularity of quitting, cutting down and temporary abstinence as reasons for using e-cigarettes is consistent with previous research (Adkison et al., 2013; Dockrell et al., 2013). Recent ex-smokers were significantly more likely than current smokers to cite taste as a reason for using an e-cigarette. It is plausible that taste would partly mediate the use of e-cigarettes given the role that olfactory and taste cues play in the behaviourally reinforcing effects of cigarettes (Perkins et al., 2001). The finding that recent ex-smokers were significantly more likely to use e-cigarettes daily than current smokers may be a reflection of ex-smokers escalating their use following cessation in the belief that the e-cigarette is an effective nicotine replacement device. To provide an indication of the ‘real-world’ effectiveness of e-cigarettes, future studies should examine the association between smoking status and the reported use of different treatments as aids to cessation among those who made a recent attempt to quit.

The popularity of E-lites identified in the current study may have arisen in part from this being the first brand to have been advertised nationally in Great Britain. From one point of view, the result is nonetheless surprising; data from convenience stores' sales figures suggest that E-lites, rather than being a clear market leader, are second to Nicolites (Eastwood, 2012). The current finding, therefore, illustrates the importance of asking smokers directly about their usage and may reflect the significance of online sales for e-cigarettes. E-lites have a visible online presence, including a range of social media pages, and are widely and easily available to purchase from online stores (Goniewicz, Knysak, et al., 2013). In terms of price, E-lites ‘starter kits’ (2 × disposable tips, 1 rechargeable battery and 1 charger) are available at approximately £25 but all leading e-cigarette brands are made more accessible by a range of obtainable discounts and incentives (Huang, Kornfield, Szczypka, & Emery, 2013). Of greater significance than price is that the nicotine delivery of E-lites is low compared with many other e-cigarette brands readily available online (Goniewicz, Kuma, Gawron, Knysak, & Kosmider, 2013; Goniewicz et al., 2014). Thus, the prominence of E-lites in Great Britain – as an e-cigarette brand that delivers a relatively small dose of nicotine – should be incorporated within future evaluations of the real-world effectiveness of e-cigarettes.

The association of usage with socio-economic status in the current study was similar to a British study at the start of 2012, which reported non-significant associations in the same direction (Dockrell et al., 2013), and international research which has reported that well educated individuals are more likely to use e-cigarettes (Adkison et al., 2013; Pearson et al., 2012). Other research suggests that as new expensive technologies emerge they are typically disproportionately adopted in the early phase by those with greater resources (Chang & Lauderdale, 2009). However, if e-cigarettes prove effective and are not to widen the health inequalities caused by smoking then communications concerning relative risks and the benefits of e-cigarettes over traditional smoking may need to be specifically targeted at more deprived groups in society.

A potential limitation of the current study is that the recruitment method is likely to have led to selection bias. The study recruited from a panel that consisted of individuals who were interested in participating in research surveys in exchange for vouchers or entering prize draws and may not be representative of the wider population. Additionally, participants were invited to take part in the study by email and completed the questionnaire online. As a result certain socio-demographic groups are likely to have been under-represented, for example both older individuals and those with lower incomes typically have fewer online skills and more limited internet access (Dutton & Blank, 2011). However, the overall sample characteristics remained broadly similar to those of representative samples obtained through a household survey (Fidler et al., 2011). Also the cross-sectional nature of the study does not permit inferences to be drawn about the causal nature of the associations found. A third limitation is that this is a snapshot in time of a phenomenon that appears to be changing rapidly. It will be important to continue to track this phenomenon over time.

## 5 Conclusions

In conclusion, there is now a near universal awareness of e-cigarettes and their use appears to be common among smokers in Great Britain although a quarter of all smokers are unsure as to whether e-cigarettes are less harmful than cigarettes. The brand E-lites, which delivers a relatively low dose of nicotine, is the most popular. E-cigarette users appear to have higher socio-economic status and to be heavier smokers who were more likely to have attempted to quit in the past year than non-users. Insofar that e-cigarettes prove to be effective in aiding smoking cessation, it appears that there will be a need to communicate the relative risks of e-cigarettes compared with traditional smoking, particularly targeted at more deprived smokers.

## Role of funding sources

The work was undertaken by the UK Centre for Tobacco and Alcohol Studies, a UKCRC Public Health Research Centre of Excellence. Funding from the Medical Research Council, British Heart Foundation, Cancer Research UK, Economic and Social Research Council and the National Institute for Health Research under the auspices of the UK Clinical Research Collaboration, is gratefully acknowledged (MR/K023195/1).

## Contributors

AM & RW conceived of the project with input on the design from JB. JB conducted the statistical analysis. JB wrote the first draft of the manuscript and RW, SM & AM all provided significant input in re-drafting. All authors contributed to and have approved the final manuscript.

## Conflict of interest

JB has received an unrestricted research grant from Pfizer. RW undertakes research and consultancy and receives fees for speaking from companies that develop and manufacture smoking cessation medications (Pfizer, J&J, McNeil, GSK, Nabi, Novartis, and Sanofi-Aventis). He also has a share of a patent for a novel nicotine delivery device (not an e-cigarette). There are no other financial relationships with any organisations that might have an interest in the submitted work, particularly electronic cigarette companies.

## Figures and Tables

**Figure:**
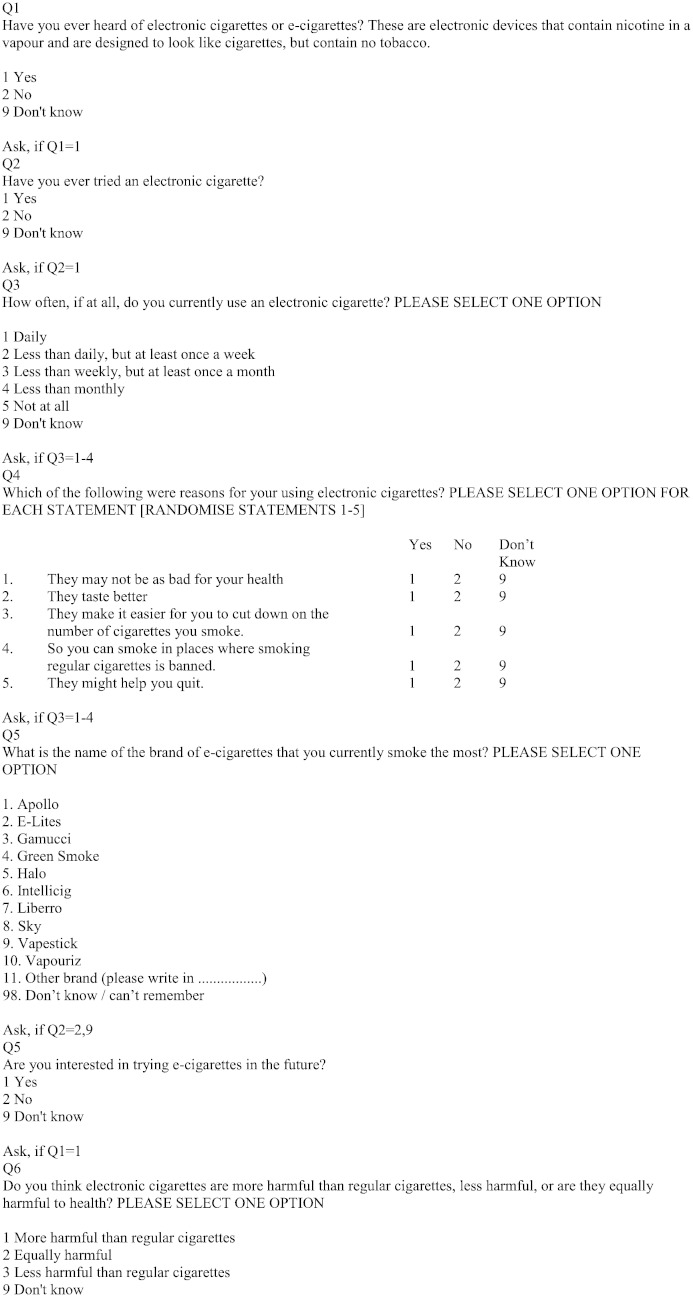


**Table 1:** Socio-demographic and smoking characteristics of the sample.

	Current smokers (n = 3538)	Recent ex-smokers (n = 579)	*P*
Mean (SD) age	43.4 (14.8)	43.7 (15.2)	NS
% (N) women	50.0 (414)	52.0 (301)	NS
% (N) high socio-economic status^a^	45.8 (1621)	54.0 (313)	***
Mean (SD) cigarettes per day	12.9 (8.8)	14.7 (10.6)	***
% quit attempt in past year	46.3 (1639)	–	–

Note: NS=non-significant, *** p < 0.001.

a ‘High’ socio-economic status includes individuals classified into managerial, professional and intermediate occupational groups by the NS-SEC.

**Table 2:** E-cigarettes: Awareness, use and beliefs by smoking status.

	Current smokers	Recent ex-smokers	*P*
**Full sample**	(**n** **=** **3538**)	(**n** **=** **579**)	
% (N) awareness	93.4 (3303)	92.9 (538)	NS
% (N) ever use	36.5 (1291)	37.3 (216)	NS
% (N) current use	21.9 (775)	18.8 (109)	NS
% (N) beliefs on harm compared with cigarettes			NS
More harmful	1.5 (52)	1.6 (9)	
Equally	7.0 (249)	9.5 (55)	
Less harmful	67.6 (2392)	63.2 (366)	
Don't know	23.9 (845)	25.7 (149)	

**Aware never users**	**(n = 2012)**	**(n = 322)**	
% (N) interest in future use	51.7 (1040)	18.3 (59)	***

**Current users**	**(n = 775)**	**(n = 109)**	
% (N) frequency of current use			***
Daily	22.7 (176)	45.9 (50)	***
Daily but ≥ once a week	36.4 (282)	19.3 (21)	**
Once a week but ≥ once a month	15.9 (123)	15.6 (17)	NS
Once a month	25.0 (194)	19.3 (21)	NS
% (N) reason for starting current use			
Health	82.6 (640)	83.5 (91)	NS
Taste	24.4 (189)	39.4 (43)	**
Cutting down	83.0 (643)	78.9 (86)	NS
Temporary abstinence	70.2 (544)	47.7 (52)	***
Quitting	82.8 (642)	84.4 (92)	NS
% (N) brand for current use			*
10 motives	1.5 (12)	0.9 (1)	NS
Apollo	1.4 (11)	0.0 (0)	NS
ClearSmoke	2.1 (16)	1.8 (2)	NS
E-cigs	1.3 (10)	0.9 (1)	NS
E-Lites	39.6 (307)	27.5 (30)	NS
Gamucci	2.2 (17)	1.8 (2)	NS
Green Smoke	3.0 (23)	2.8 (3)	NS
Halo	3.0 (23)	3.7 (4)	NS
Intellicig	2.1 (16)	2.8 (3)	NS
Liberro	1.3 (10)	0.0 (0)	NS
Nicolites	1.2 (9)	2.8 (3)	NS
Sky	7.4 (57)	3.7 (4)	NS
Vapestick	2.3 (18)	2.8 (3)	NS
Vapouriz	1.7 (13)	2.8 (3)	NS
VIP	2.3 (18)	5.5 (6)	NS
Other brand^a^	8.4 (65)	19.3 (21)	**
Don't know/can't remember	19.4 (150)	21.1 (23)	NS

NS = non-significant, * p < .05, **p < .01, *** p < 0.001.

a Other brands include all brands identified by less than 1% of the total sample.

**Table 3:** Characteristics of e-cigarette users.

	Current smokers			Recent ex-smokers		
	Currently use (n = 775)	No current use (n = 2763)	OR (95%CI) Adj. OR (95%CI)	Current use (n = 109)	No current use (n = 470)	OR (95%CI) Adj. OR (95%CI)
Mean (SD) age	42.9 (14.9)	43.5 (14.8)	1.00 (0.99–1.00) 1.00 (0.99–1.01)	45.0 (14.8)	43.4 (15.3)	1.01 (0.99–1.02) 1.00 (0.99–1.02)
% (N) women	48.1 (373)	49.7 (1373)	0.94 (0.80–1.10) 0.96 (0.81–1.13)	48.6 (53)	52.8 (248)	0.85 (0.56–1.29) 0.95 (0.61–1.46)
% (N) high socio-economic status^a^	52.9 (410)	43.8 (1211)	1.44 (1.23–1.69)*** 1.48 (1.25–1.75)***	52.3 (57)	54.5 (256)	0.92 (0.60–1.39) 0.99 (0.64–1.54)
Mean (SD) cigarettes per day	13.9 (8.9)	12.6 (8.7)	1.02 (1.01–1.02)** 1.02 (1.01–1.03)***	17.5 (10.9)	14.0 (10.5)	1.03 (1.01–1.05)** 1.03 (1.01–1.05)**
% quit attempt in past year	31.7 (246)	21.8 (601)	2.81 (2.38–3.32)*** 2.82 (2.38–3.34)***	–	–	–

Note: The adjusted models include all the other socio-demographic and smoking variables presented in Table 3. **p < .01, *** p < 0.001.

a ‘High’ socio-economic status includes individuals classified into managerial, professional and intermediate occupational groups by the NS-SEC.
